# Procalcitonin/albumin to urea nitrogen ratio: a novel prognostic indicator for severe fever with thrombocytopenia syndrome

**DOI:** 10.1186/s12879-026-12736-6

**Published:** 2026-01-29

**Authors:** Song Li, Bing Gao, Lei Tian, Qing Tang, Xu Xiang

**Affiliations:** Department of Laboratory Medicine, Tongji Hospital, Tongji Medical College, Huazhong University of Science and Technology, Wuhan, 430030 China

**Keywords:** Procalcitonin, Blood urea nitrogen, Albumin, Severe fever with thrombocytopenia syndrome, Prognosis

## Abstract

**Objective:**

Severe fever with thrombocytopenia syndrome (SFTS), caused by the novel Bunyavirus (NBV), is a tick-borne infectious disease associated with high morbidity and mortality. Currently, no effective treatment or vaccine exists for SFTS. Early identification of prognostic factors is crucial for clinical management. This study aimed to evaluate the prognostic value of the procalcitonin/albumin to urea nitrogen ratio (PAU) in SFTS patients.

**Methods:**

This retrospective observational study enrolled 259 consecutive patients with SFTS admitted to Tongji Hospital from April 2023 to November 2024. Patients were stratified into survival (*n* = 172) and death (*n* = 87) groups based on clinical outcomes.

**Results:**

Multivariable logistic regression identified advanced age, altered mental status (AMS), elevated PAU and high viral load as independent predictors of adverse outcome in SFTS. PAU exhibited strong discriminative performance (AUC 0.810; 95% CI 0.757–0.857; sensitivity 75.7%, specificity 73.0%), with an optimal cut-off of 0.06 µmol/L. Integration of PAU with age, viral load and AMS further improved accuracy (AUC 0.897; 95% CI 0.858–0.936). Bootstrap internal validation yielded a calibration curve closely approximating the diagonal, confirming robust predictive efficacy of the composite model.Kaplan-Meier analysis revealed significantly worse prognosis in high-PAU patients (*P* < 0.001).The longitudinal trend analysis revealed an increasing trend in PAU among non-survivors, while survivors exhibited a decreasing trend.

**Conclusion:**

PAU represents a novel composite biomarker that integrates inflammatory and organ dysfunction parameters, providing reliable prognostic prediction for SFTS patients.

**Clinical trial number:**

Not applicable.

**Supplementary Information:**

The online version contains supplementary material available at 10.1186/s12879-026-12736-6.

## Introduction

Severe Fever with Thrombocytopenia Syndrome (SFTS) is an acute zoonotic infectious disease caused by the novel Bunyavirus (NBV). The primary clinical manifestations include leukopenia and thrombocytopenia, accompanied by systemic symptoms such as fever, fatigue, myalgia, and gastrointestinal disturbances, including diarrhea and vomiting. A small proportion of patients experience rapid disease progression, ultimately leading to multiorgan failure with a reported mortality rate of up to 30% [1–2]. Since its initial identification in China in 2009, SFTS due to NBV has garnered global attention due to its high morbidity and mortality [3]. In 2017, the World Health Organization (WHO) designated SFTS as one of the diseases requiring urgent research prioritization [4]. Given the rapid progression and high mortality associated with severe cases, early identification of reliable prognostic indicators is crucial for effective clinical management.

Previous studies have identified several biomarkers with potential prognostic value for SFTS, including the Neutrophil-to-Lymphocyte Ratio (NLR), Platelet-to-Lymphocyte Ratio (PLR), Platelet-to-Albumin Ratio (PAR), and AST/ALT ratio [5–8]. However, the diagnostic performance of these markers varies across studies due to differences in selected indicators and datasets, resulting in inconsistent predictive accuracy. A recent study proposed a multi-parameter scoring system for prognostic evaluation; however, its complexity limits practical clinical application [9]. Therefore, the development of simpler and more robust composite biomarkers is of significant importance.

Evidence suggests that multiorgan dysfunction and systemic inflammatory responses are key contributors to poor outcomes in SFTS patients [10–11]. NBV infection can cause extensive organ damage, with Blood Urea Nitrogen (BUN) and serum Albumin (ALB) serving as indicators of hepatic and renal impairment. A recent study demonstrated that the Blood Urea Nitrogen-to-Albumin Ratio (BAR) has promising prognostic utility in SFTS [12]. However, these investigations primarily focused on organ dysfunction as a singular dimension and did not incorporate inflammatory parameters into their analyses.

Inflammatory cytokine storm is a major pathogenic mechanism in severe SFTS, and Procalcitonin (PCT), a biomarker reflecting the severity of systemic inflammation, has been identified as an independent predictor of mortality in SFTS patients [13]. Therefore, combining PCT with BAR may provide a more comprehensive assessment of prognosis by integrating both organ dysfunction and inflammatory response, potentially enhancing predictive accuracy. To the best of our knowledge, no prior studies have evaluated the prognostic role of PAU in SFTS patients. Hence, this study aims to investigate whether PAU can serve as an early risk stratification tool for SFTS and whether it outperforms existing prognostic indicators.

## Patients and methods

### Criteria for patient inclusion and exclusion

This retrospective observational study included a total of 259 patients diagnosed with SFTS at Tongji Hospital in Wuhan between April 2023 and November 2024. Inclusion criteria: (1) age ≥ 18 years; (2) documented epidemiological exposure; (3) positive NBV nucleic-acid test.Exclusion criteria: (1) incomplete admission laboratory data, specifically missing PCT, BUN, or ALB; (2) hospitalization < 24 h; (3) pre-existing chronic liver or kidney disease that could influence baseline PAU values; (4) co-infection with another pathogen when SFTS was not the primary diagnosis.

The study protocol followed the Declaration of Helsinki and was approved by the Institutional Ethics Committee of Tongji Hospital, Huazhong University of Science and Technology (Approval No. TJ-IRB202411077). Informed consent was waived because of the retrospective design.

**Fig. 1:**
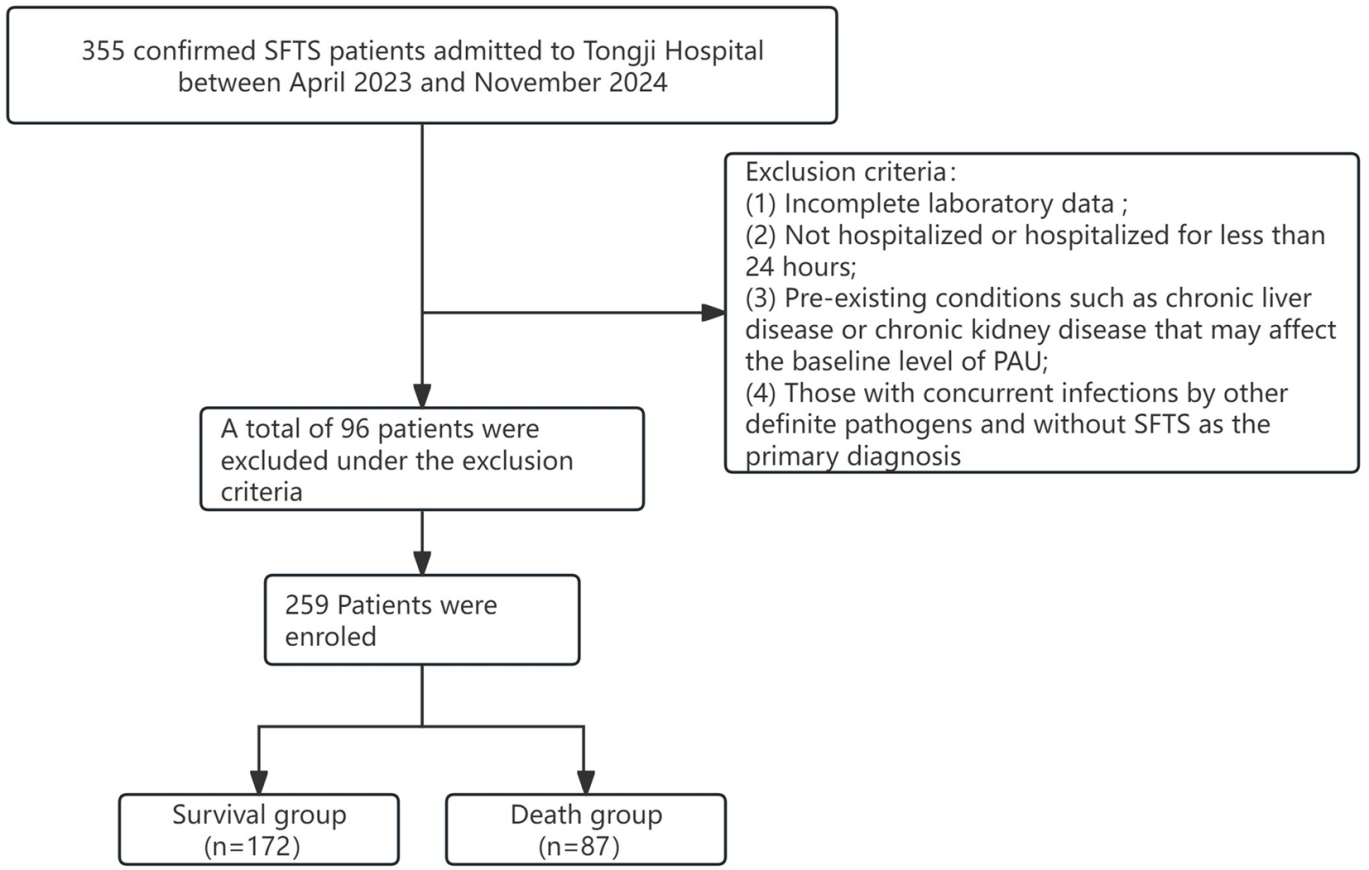
Flowchart of patient screening

Of 355 confirmed SFTS cases initially identified, 96 were excluded: 40 for incomplete data, 9 for hospitalization < 24 h, 25 for chronic liver disease, 8 for chronic kidney disease, and 14 for other active infections. The remaining 259 patients constituted the analytic cohort. In this study, 40 patients (11.3%) with missing data were excluded. No significant systematic bias was found between the excluded and included populations in baseline characteristics such as age and sex. NBV RNA was detected with the hospital’s standardized molecular platform. Death endpoints were independently reviewed by two senior physicians to ensure consistency.See Fig. 1.

### Data collection and categorization

We systematically collected comprehensive baseline information and laboratory data from the hospital’s electronic medical record system upon patient admission. Additionally, we gathered both the initial blood tests performed at admission and follow-up blood tests conducted prior to discharge or dying to enable dynamic subgroup analysis and evaluate temporal trends of PAU. The clinical dataset encompassed key demographic and clinical characteristics(gender, age, clinical outcomes, temperature records, days from onset to diagnosis, length of stay), as well as underlying comorbidities (diabetes mellitus [DM], hypertension [HTN], coronary heart disease [CHD], cerebrovascular disease [CVD], autoimmune disease[AIDs], chronic obstructive pulmonary disease[COPD], cancer[CA]), clinical symptoms (fatigue, diarrhea, vomiting, muscle soreness, altered mental status), and laboratory parameters (white blood cell count [WBC], neutrophil count [NEUT], lymphocyte count [LYM], platelet count [PLT], serum albumin [ALB], alanine aminotransferase [ALT], aspartate aminotransferase [AST], lactate dehydrogenase [LDH], blood urea nitrogen [BUN], procalcitonin [PCT], activated partial thromboplastin time [APTT], prothrombin time [PT], and NBV viral load). All data were entered into a standardized Excel database and independently verified by two trained investigators. The following composite biomarkers were calculated: PAU = PCT × BUN / ALB; NLR = NEUT / LYM; PLR = PLT / LYM; PAR = PLT / ALB; BAR = BUN / ALB.

Altered mental status (AMS) was defined as the presence of any of the following neurological manifestations: confusion, disorientation, lethargy, unresponsiveness, or coma.

NBV RNA Detection: NBV RNA was extracted on the Xi’an Tianlong PANA9600s and amplified with the GENTIER 96E system using Da’an Gene (Sun Yat-sen University) kits, following the manufacturer’s protocol.The linear detection range was 1.0 × 10² to 1.0 × 10⁶ TCID50/mL, with a conversion factor of 1 TCID50/mL = 496 copies/mL.

**Clinical outcome**: ① The endpoint of observation was 28 days after admission. During this period, patients who died were classified as the death group (87 cases), and those who survived were classified as the survival group (172 cases). ② Immediate causes of death were adjudicated independently by two senior physicians: multiorgan dysfunction syndrome (56, 64.4%), severe hemorrhage (15, 17.2%), central nervous system complications (10, 11.5%), and septic shock (6, 6.9%).

**Treatment protocols** : ①Invasive mechanical ventilation, continuous pumping of catecholamines, and continuous renal replacement therapy (CRRT) were confined to the intensive care unit (ICU); general wards provided basic monitoring, oxygen, and non-invasive antihypertensives.② Antiviral therapy: ribavirin within 24 h of admission; short-course methylprednisolone when indicated.③ Secondary bacterial infection was managed empirically with piperacillin–tazobactam or meropenem, then adjusted according to culture results and resistance profiles.④ Psychological support: patients with delirium or mood disturbances were assessed by the psychiatry team.

#### Secondary infection surveillance

During the inclusion stage, cases of “co-infection with another pathogen when SFTS was not the primary diagnosis” were excluded. During hospitalization, dynamic blood, sputum, urine cultures, serum G/GM tests and common viral nucleic acid tests are conducted. Secondary infection was recorded only when new infectious foci were microbiologically and clinically confirmed.

### Statistical methods

Statistical analyses were conducted using IBM SPSS Statistics (version 25.0) and R statistical software (version 4.4.1). The Shapiro-Wilk test was used to assess data normality. Normally distributed continuous variables were expressed as mean±standard deviation (SD), while non-normally distributed variables were presented as median and interquartile range (IQR). Comparisons between groups were performed using the independent samples t-test for normally distributed data and the Mann-Whitney U test for skewed distributions. Categorical variables were analyzed using the chi-square (χ²) test. A P-value < 0.05 was considered statistically significant.

To mitigate multicollinearity, we calculated variance inflation factors (VIFs) before entering variables into the multivariable logistic model and excluded any with VIF > 5.Univariate logistic regression analysis was performed to identify potential prognostic factors associated with mortality. Variables with *P* < 0.05 in univariate analysis were further included in multivariate logistic regression to determine independent predictors. Receiver Operating Characteristic (ROC) curve analysis was employed to evaluate the predictive accuracy of each biomarker for in-hospital mortality. The optimal cutoff value was determined using the Youden index to maximize the sum of sensitivity and specificity.Internal validation was performed with 1,000 bootstrap resamples. Model performance was assessed using the C-index, calibration curve, and ROC curve. A two-sided P value < 0.05 was considered statistically significant.Kaplan-Meier survival analysis was used to estimate cumulative survival probabilities in patients stratified by PAU levels, and differences between groups were assessed using the log-rank test. Spearman correlation analysis was conducted to evaluate the association between PAU and other clinical parameters. Correlation strength was interpreted as follows: 0.2 < *r* ≤ 0.3 (weak), 0.3 < *r* ≤ 0.6 (moderate), 0.6 < *r* ≤ 0.8 (strong), and *r* > 0.8 (very strong), with statistical significance set at *P* < 0.05.

## Results

### Demographic, Clinical, and laboratory characteristics of patients with SFTS

A total of 259 SFTS patients were enrolled; 172 survived and 87 died (mortality 33.59%). Compared with the survival group, the ICU transfer rate, invasive mechanical ventilation, continuous pumping of catecholamines and CRRT in the death group were significantly increased (Table S1). Secondary infections, empirical antibiotic use and psychological consultation increased simultaneously (Table S2). The bacteria most frequently identified were Acinetobacter baumannii, Klebsiella pneumoniae and Staphylococcus aureus. In the death group, two cases of fungal infection were also observed (one case of Candida albicans bloodstream infection and one case of aspergillus pneumonia), and no other new viruses were detected.

In terms of clinical features, patients in the death group were older and had a higher proportion of CHD, CVD and AMS(all *P* < 0.05).Laboratory profiling revealed a multi-system injury signature in the death group: liver (ALT, AST), kidney (BUN), myocardial (LDH), coagulation (APTT, PT) and inflammatory (PCT, PAU) indices were all markedly elevated accompanied by higher viral load, whereas LYM, PLT and ALB levels were significantly reduced (all *P* < 0.001). See Table 1.

**Table 1:** Comparison of demographic, clinical, and laboratory characteristics between the survival group and the death group in SFTS patients

Characteristics	Survival(*n* = 172)	Death (*n* = 87)	t/z/χ² value	*P* Value
Age (year)	63.0(55.0,70.0)	70.0(64.0,75.0)	-4.887	< 0.001
Gender(male), n (%)	75(43.60)	48(55.17)	3.100	0.078
Underlying diseases, n (%)				
DM, n (%)	19(11.04)	13(14.94)	0.810	0.368
HTN, n (%)	56(32.55)	36(41.37)	1.963	0.161
CHD, n (%)	8(4.65)	11(12.64)	5.429	0.020
CVD, n (%)	9(5.23)	16(18.39)	11.471	< 0.001
AIDs, n (%)	3(1.74)	2(2.29)	0.094	0.759
COPD, n (%)	7(4.07)	5(5.74)	0.368	0.544
CA, n (%)	5(2.90)	4(4.59)	0.492	0.483
Clinical symptoms, n (%)				
Muscle soreness, n (%)	32(18.60)	12(13.79)	0.948	0.330
Fatigue, n (%)	168(97.67)	87(100.00)	0.810	0.368
Diarrhoea, n (%)	79(45.93)	49(56.32)	2.496	0.114
Vomiting , n (%)	41(23.83)	28(32.18)	2.060	0.151
AMS, n (%)	26(15.11)	44(50.57)	36.832	< 0.001
Body temperature (℃)	36.90(36.50,37.50)	36.90(36.50,38.00)	-0.644	0.519
Days from onset to Admission(day)	7.00(5.00,8.00)	7.00(5.00,7.00)	1.091	0.266
Laboratory indicators (normal range)				
WBC(3.5–9.5 × 10^9^/L)	3.60(2.34,5.81)	3.43(2.28,6.01)	0.360	0.719
NEUT(1.8–6.3 × 10^9^/L)	2.42(1.18,4.16)	2.31(1.31,4.14)	-0.538	0.591
LYMPH(1.1–3.2 × 10^9^/L)	0.75(0.39,1.24)	0.59(0.33,0.94)	1.999	0.046
PLT (125–350 × 10^9^/L)	52.0(38.0,70.0)	37.0(26.0,54.0)	4.112	< 0.001
ALB (40–55 g/L)	33.40(30.50,37.10)	32.20(29.30,34.20)	2.982	0.003
ALT (< 40 U/L)	75.0(42.0,136.0)	118.0(65.0,211.0)	-3.750	< 0.001
AST (< 35 U/L)	169.0(87.0,347.0)	404.0(213.0,687.0)	-6.076	< 0.001
AST/ALT	2.30(1.69,3.11)	3.28(2.63,4.06)	-5.987	< 0.001
LDH (120–250 U/L)	602.0(377.0,946.0)	1220.0(797.0,1752.0)	-6.404	< 0.001
BUN(3.1–8.0 mmol/L)	5.40(3.90,6.80)	8.70(6.00,12.80)	-6.689	< 0.001
PCT(<0.05ng/ml)	0.16(0.09,0.40)	0.61(0.25,1.82)	-7.163	< 0.001
PAU*	0.03(0.01,0.06)	0.15(0.06,0.65)	-7.952	< 0.001
APTT(29–42 s)	49.10(40.50,60.80)	61.30(52.70,75.90)	-5.966	< 0.001
PT (11.5–14.5 s)	12.60(11.90,13.10)	13.10(12.40,13.70)	-4.223	< 0.001
Viralload(log_10_copies/mL)	5.79(4.70,6.58)	7.30(6.48,8.16)	-8.051	< 0.001

Abbreviations: DM, diabetes; HTN, hypertension; CHD, coronary heart disease; CVD: cerebrovascular disease; AIDs, Autoimmune disease; COPD, Chronic obstructive pulmonary disease; CA, Cancer; AMS, altered mental status; WBC, white blood cell. NEUT, neutrophil count; LYMPH, lymphocyte count; PLT, platelet; ALB, albumin; ALT, alanine aminotransaminase; AST, aspartate aminotransferase; LDH, lactate dehydrogenase; BUN, blood urea nitrogen; PCT, procalcitonin ; PAU : procalcitonin /albumin to urea ratio; APTT, activated partial thromboplastin time; PT, prothrombin time; SFTS, severe fever with thrombocytopenia syndrome

### Multicollinearity check

Before fitting the multivariable model we computed variance-inflation factors (VIFs) for all candidate predictors. Because PAU is a linear composite of PCT, BUN and ALB, only PAU was retained to avoid perfect collinearity. AST/ALT ratio was used instead of separate AST and ALT values, and all other variables that differed significantly in Table 1 were entered into the VIF analysis. The highest VIF observed was 2.67 (all values < 5),suggesting no significant multicollinearity.

### Univariate and multivariate analyses of the prognosis of SFTS patients

Univariate and multivariable analyses were performed with the above 12 variables as independent variables and clinical outcomes as the dependent variables. Four independent risk factors were identified: Age (odds ratio [OR]: 1.09; 95% confidence interval [CI]: 1.04–1.14; *P* < 0.001), AMS (OR: 2.50; 95% CI: 1.10–5.71;*P* = 0.028), PAU (OR: 14.14; 95% CI: 3.16–54.87; *P* = 0.002), and viral load (OR: 3.05; 95% CI: 2.17–4.49; *P* < 0.001). See Table 2.

**Table 2:** Univariate and multivariate logistic regression analysis for the prognosis of SFTS patients

Variables	Univariate		Multivariate	
	OR (95%CI)	*P* Value	OR (95%CI)	*P* Value
Age	1.07(1.04–1.10)	<0.001	1.09(1.04–1.14)	<0.001
CHD	2.96(1.14–7.67)	0.025		
CVD	4.08(1.72–9.67)	0.001		
AMS	5.74(3.17–10.38)	<0.001	2.50(1.10–5.71)	0.028
LYMPH	0.81(0.58–1.14)	0.243		
PLT	0.97(0.96–0.99)	<0.001		
AST/ALT	1.76(1.40–2.20)	<0.001		
LDH	1.01(1.01–1.01)	<0.001		
PAU	35.34(7.70-162.17)	<0.001	14.14 (3.16–54.87)	0.002
APTT	1.04(1.02–1.05)	<0.001		
PT	1.72(1.34–2.19)	<0.001		
Viral load	3.01(2.24–4.02)	<0.001	3.05(2.17–4.49)	<0.001

Abbreviations: OR, odds ratio; CI, confidence interval; CHD, coronary heart disease; CVD, cerebrovascular disease; AMS, altered mental status; LYMPH, lymphocyte count; PLT, platelet; ALT, alanine aminotransaminase; AST, aspartate aminotransferase; LDH, lactate dehydrogenase; PAU, procalcitonin /albumin to urea ratio; APTT, activated partial thromboplastin time; PT, prothrombin time; SFTS, severe fever with thrombocytopenia syndrome

### Diagnostic performance of PAU for distinguishing the death group from the survival group in SFTS patients

**Fig. 2:**
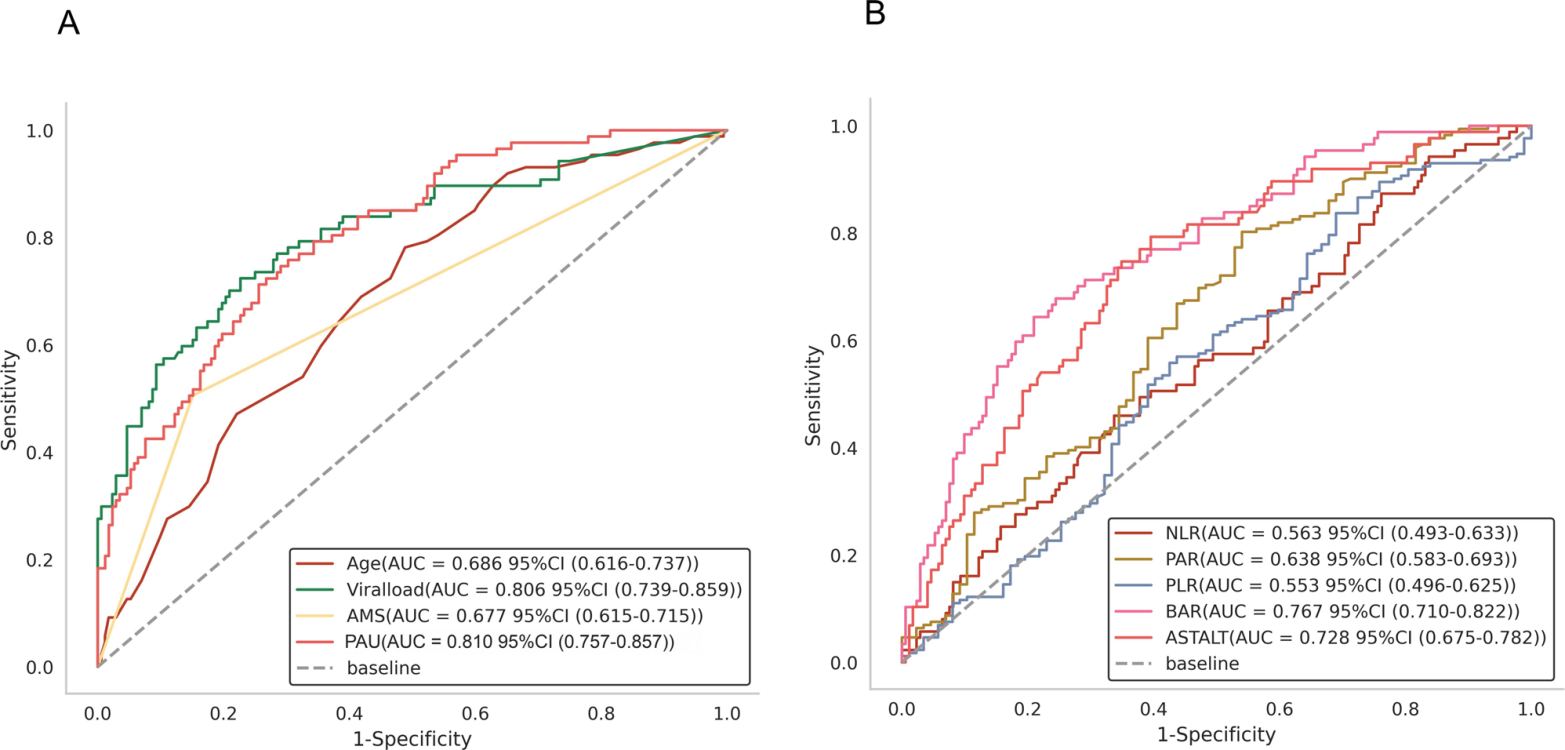
ROC Curve Analysis (**A**) Prognostic analysis of SFTS patients with viral load, AGE, AMS, and PAU. (**B**) Prognostic analysis of SFTS patients with NLR, PLR, PAR, AST/ALT, and BAR

To determine the diagnostic performance of PAU for fatal outcomes in patients with SFTS, the predictive efficiency of PAU was compared with that of the above-mentioned several independent risk factors. The area under the curve (AUC) of PAU (0.810; 95% CI 0.757–0.857; sensitivity 75.7%, specificity 73.0%) exceeded those of Age, AMS and Viral load, as shown in Fig. 2A. In addition, PAU was compared with several other classic indicators. PAU is more capable of predicting the poor prognosis of SFTS patients at admission than NLR, PLR, PAR, AST/ALT and BAR, and has more reliable predictive performance, as shown in Fig. 2B. The optimal cut-off value of PAU determined by the maximum Youden index was 0.06 µmol/L ( Table 3).

**Table 3:** Diagnostic performances of prognostic indicators for distinguishing the death group from the survival group in SFTS patients

Variables	AUC (95%CI)	Sensitivity	Specificity	Youden’s index	Cut off
Age	0.686(0.616–0.737)	78.2%	51.2%	0.293	64.0
AMS	0.677(0.615–0.715)	50.6%	84.9%	0.355	1.00
PAU	0.810(0.757–0.857)	75.7%	73.0%	0.487	0.06
Viral load	0.806(0.739–0.859)	72.4%	77.3%	0.497	6.66
NLR	0.563(0.493–0.633)	46.0%	66.3%	0.123	4.81
PLR	0.553(0.496–0.625)	83.7%	31.0%	0.148	34.69
PAR	0.638(0.583–0.693)	80.2%	46.0%	0.262	1.05
AST/ALT	0.728(0.675–0.782)	74.7%	65.1%	0.398	2.63
BAR	0.767(0.710–0.822)	64.4%	79.1%	0.434	0.22

Abbreviations: AUC, area under the curve; CI, confidence interval; AMS, altered mental status; PAU, procalcitonin/albumin to urea ratio; NLR, neutrophil to lymphocyte ratio; PLR, platelet to lymphocyte ratio; PAR, platelet to albumin ratio; ALT, alanine aminotransaminase; AST, aspartate aminotransferase; BAR, blood urea nitrogen to albumin ratio; SFTS, severe fever with thrombocytopenia syndrome

### Prognostic model incorporating PAU for mortality risk prediction

**Fig. 3:**
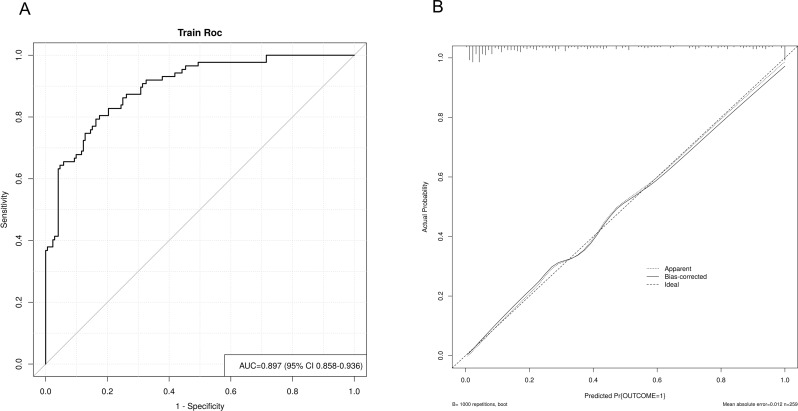
Multivariable logistic model and calibration curve predicting prognostic risk for SFTS patients (**A**) ROC curve of the model incorporating age, viral load, PAU, and AMS; the AUC is 0.897 (95% CI 0.858–0.936). (**B**) Calibration plot based on 1,000 bootstrap resamples, with a mean absolute error of 0.012, indicating excellent agreement between predicted and observed probabilities

Using the four independent predictors identified by multivariable logistic regression, we developed a prognostic model for SFTS mortality: logit(P) = − 14.469 + 0.087 × Age + 1.117 × Viral load + 2.650 × PAU + 0.917 × AMS.The model achieved an AUC of 0.897 (95% CI 0.858–0.936), with 79.3% sensitivity, 83.7% specificity and 81.5% overall accuracy (Fig. 3A). Bootstrap validation (1 000 resamples) yielded a calibration slope indistinguishable from the diagonal (mean absolute error [MAE] = 0.012), confirming excellent agreement between predicted and observed probabilities (Fig. 3B).

### Correlation between PAU and laboratory parameters

At admission, there were significant differences (*P* < 0.05) between the death group and the survival group in terms of liver function, cardiac function, renal function, inflammatory indicators, coagulation function and viral load. Correlation analysis showed that PAU was highly correlated with PCT (*r* = 0.942) and highly correlated with BUN (*r* = 0.692). It was moderately correlated with LDH (*r* = 0.573), AST (*r* = 0.460), Viral load (*r* = 0.508), APTT (*r* = 0.438), and ALT (*r* = 0.307), and weakly correlated with PT (*r* = 0.257). In addition, PAU was moderately negatively correlated with PLT (*r* = -0.379) and ALB (*r* = -0.363). These data demonstrate that PAU integrates information on inflammation, multiorgan injury and viral replication, validating its utility as a composite marker of disease severity in SFTS. The correlation analyses are displayed in Table 4.

**Table 4:** Correlation between PAU and laboratory parameters in SFTS patients

			PCT	BUN	LDH	AST
Spearman’s rho	PAU	Correlation Coefficient	0.942	0.692	0.573	0.460
		Sig. (2-tailed)	<0.001	<0.001	<0.001	<0.001

	Viral load	APTT	ALT	PT	PLT	ALB	
Correlation Coefficient	0.508	0.438	0.307	0.257	-0.379	-0.363	
Sig. (2-tailed)	<0.001	<0.001	<0.001	<0.001	<0.001	<0.001	

Abbreviations: PAU, procalcitonin/albumin to urea ratio; PCT, procalcitonin ; BUN, blood urea nitrogen; LDH, lactate dehydrogenase; AST, aspartate aminotransferase; APTT, activated partial thromboplastin time; ALT, alanine aminotransaminase; PT, prothrombin time; PLT, platelet; ALB, albumin; SFTS, severe fever with thrombocytopenia syndrome

### Comparison of baseline characteristics between high and low PAU groups

**Fig. 4:**
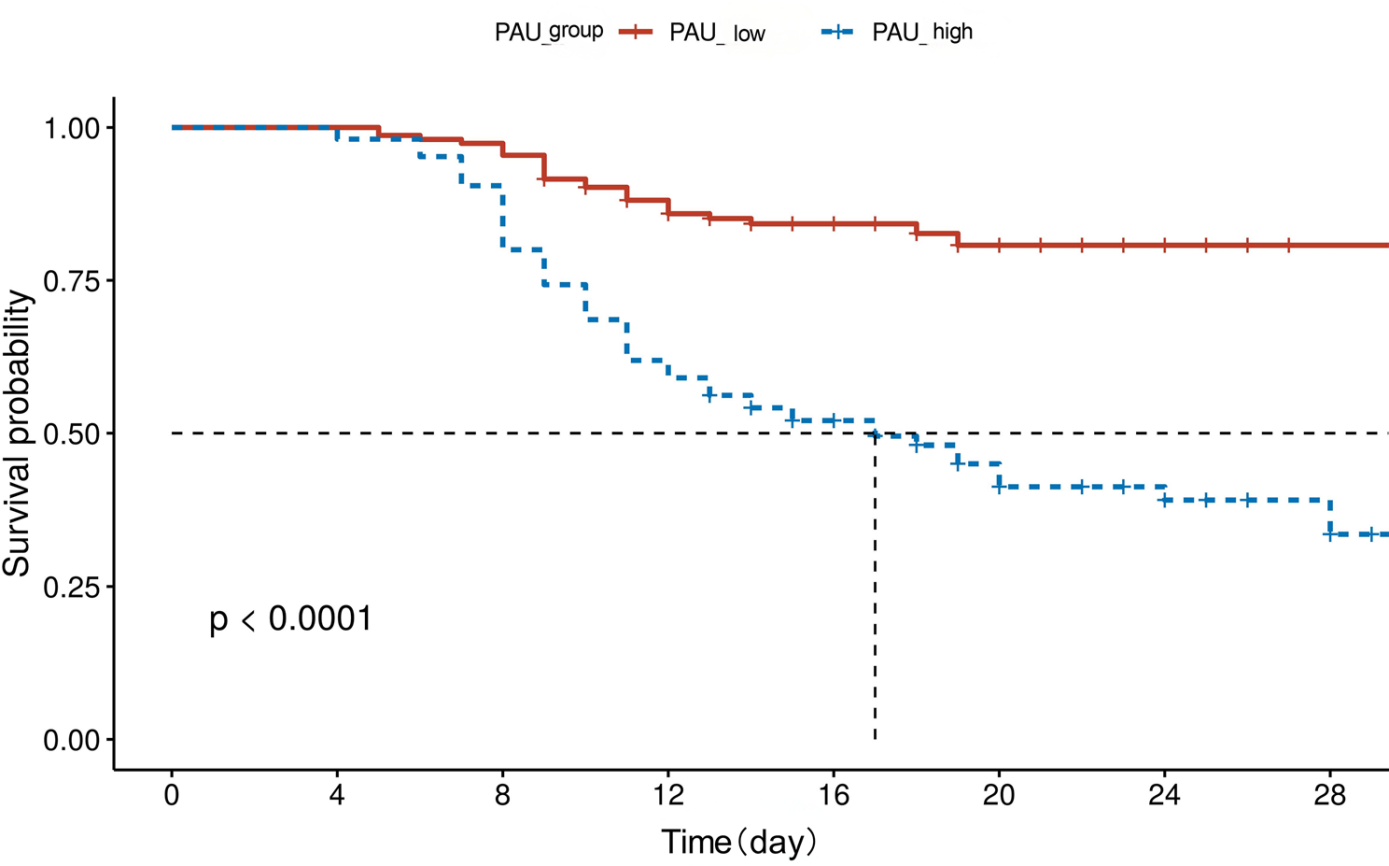
Kaplan–Meier 28-day survival curves stratified by the optimal PAU cut-off of 0.06 µmol/L. The difference in cumulative survival between the low-PAU and high-PAU groups was assessed by the log-rank test (*P* < 0.001)

According to the results of multivariate logistic regression analysis, PAU was identified as an independent risk factor for mortality in patients with SFTS. Using a cutoff value of 0.06µmol/L, patients were stratified into low PAU and high PAU groups. Analysis of clinical characteristics revealed that patients in the high PAU group were older, had a higher proportion of males, and exhibited a significantly higher mortality rate. Additionally, the prevalence of HTN, CVD, and AMS was significantly elevated in this group. Regarding laboratory parameters, patients with high PAU levels demonstrated more pronounced organ dysfunction and a heightened inflammatory response. Specifically, liver function (ALT, AST), myocardial injury (LDH), renal function (BUN), coagulation abnormalities (APTT, PT), inflammatory activity (PCT), and viral load were all significantly higher in the high PAU group compared to the low PAU group. In contrast, PLT and ALB levels were significantly lower. Notably, no statistically significant differences were observed in WBC, NEUT, or LYM between the two groups, as presented in Table 5. To further assess the prognostic value of PAU, Kaplan-Meier survival analysis was performed. The results revealed that the 28-day cumulative survival rate was significantly higher in the low PAU group than in the high PAU group (*P* < 0.001), further supporting the strong association between elevated PAU levels and adverse clinical outcomes in SFTS patients, as illustrated in Fig. 4.

**Table 5:** Comparison of baseline characteristics between the low PAU group and the high PAU group in SFTS patients

Age (year)	PAU^low^(*n* = 154)	PAU^high^ (*n* = 105)	t/z/χ² value	*P* Value	
	65.0(56.0,70.0)	68.0(59.0,75.0)	-2.406	0.016	
Mortality rate, n(%)	26(16.88)	61(58.09)	47.535	< 0.001	
Gender(male), n (%)	65(42.20)	58(55.23)	4.251	0.039	
Underlying diseases, n (%)					
DM, n (%)	15(9.74)	17(16.19)	2.399	0.121	
HTN, n (%)	44(28.57)	48(45.71)	8.011	0.005	
CHD, n (%)	9(5.84)	10(9.52)	1.244	0.265	
CVD, n (%)	10(6.49)	15(14.28)	4.347	0.037	
Clinical symptoms, n (%)					
Muscle soreness, n (%)	30(19.48)	14(13.33)	1.673	0.196	
Fatigue, n (%)	150(97.40)	105(100.00)	1.325	0.250	
Diarrhea, n (%)	73(47.40)	55(52.38)	0.619	0.431	
Vomiting , n (%)	37(24.02)	32(30.47)	1.329	0.249	
AMS, n (%)	24(15.58)	46(43.81)	25.219	< 0.001	
Laboratory indicators (normal range)					
WBC(3.5–9.5 × 10^9^/L)	3.55(2.06,5.68)	3.65(2.42,6.22)	-1.173	0.241	
NEUT(1.8–6.3 × 10^9^/L)	2.31(1.11,4.00)	2.58(1.39,4.25)	-1.368	0.171	
LYMPH(1.1–3.2 × 10^9^/L)	0.74(0.37,1.10)	0.65(0.38,1.37)	-0.273	0.786	
PLT (125–350 × 10^9^/L)	52.0(39.0,73.0)	39.0(26.0,55.0)	4.562	< 0.001	
ALB (40–55 g/L)	34.36 ± 4.35	31.69 ± 4.13	4.918	< 0.001	
ALT (< 40 U/L)	73.0(40.0,147.0)	108.0(63.0,195.0)	-3.369	< 0.001	
AST (< 35 U/L)	161.0(85.0,362.0)	325.0(202.0,655.0)	-5.476	< 0.001	
LDH (120–250 U/L)	560.0(373.0,884.0)	1191.0(751.0,1752.0)	-6.988	< 0.001	
BUN (3.1–8.0 mmol/L)	5.10(3.90,6.10)	9.20(6.50,13.80)	-9.442	< 0.001	
PCT(<0.05ng/ml)	0.13(0.08,0.21)	0.88(0.49,1.81)	-12.921	< 0.001	
APTT(29–42 s)	49.50(41.80,58.90)	61.30(49.30,75.90)	-5.094	< 0.001	
PT (11.5–14.5 s)	12.50(11.90,13.20)	13.00(12.40,13.80)	-3.622	< 0.001	
Viralload(log_10_copies/mL)	5.74(4.69,6.62)	6.92(5.98,7.65)	-6.311	< 0.001	

Abbreviations: PAU: Procalcitonin /albumin to urea ratio; DM, Diabetes; HTN, Hypertension; CHD, Coronary heart disease; CVD: Cerebrovascular disease; AMS, Altered Mental Status; WBC, white blood cell. NEUT, Neutrophil count; LYMPH, Lymphocyte count; PLT, platelet; ALB, albumin; ALT, alanine aminotransaminase; AST, aspartate aminotransferase; LDH, lactate dehydrogenase; BUN, blood urea nitrogen; PCT, Procalcitonin ; APTT, activated partial thromboplastin time; PT, prothrombin time; SFTS, severe fever with thrombocytopenia syndrome

### Association between increased PAU and poor prognosis of SFTS

**Fig. 5:**
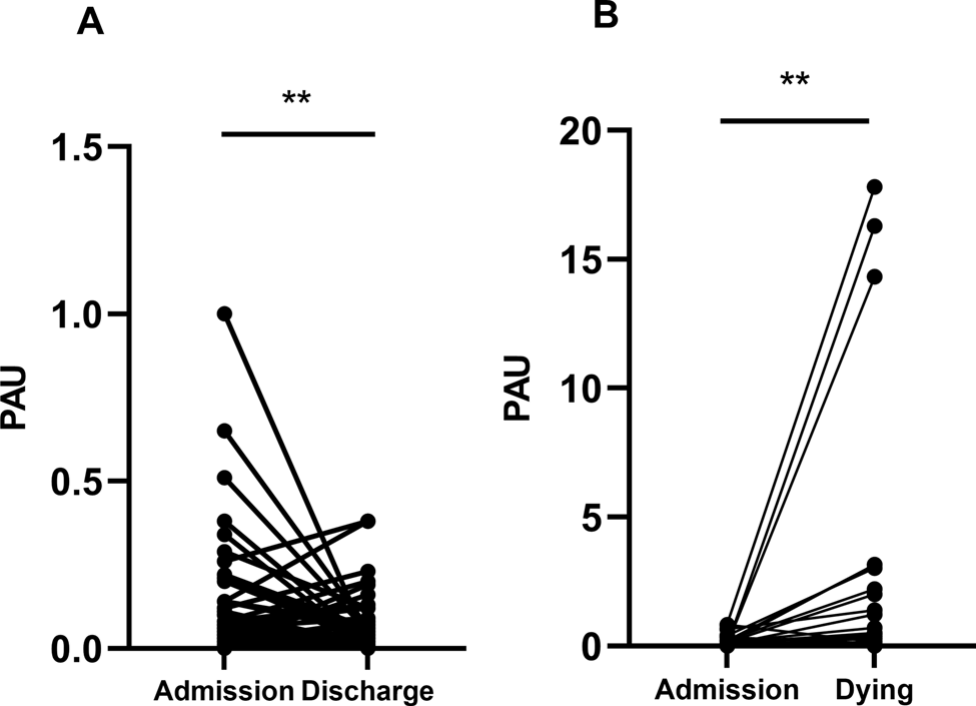
Dynamic changes of PAU levels from admission to final hospital sampling. (**A**)(survival group, *n* = 93): PAU levels declined markedly from admission to the final blood test at discharge. (**B**)(death group, *n* = 32): PAU levels rose significantly between admission and the last blood draw obtained before death.Paired comparisons were performed using the Wilcoxon signed-rank test

To further elucidate the relationship between PAU levels and the prognosis of SFTS patients, we longitudinally monitored the dynamic changes in PAU levels during the initial and final blood tests throughout hospitalization in a subset of patients from both the survival and death groups. The results revealed a distinct trend: in the death group (*n* = 32), PAU levels progressively increased with disease deterioration. Conversely, in the survival group (*n* = 93), PAU levels significantly decreased as clinical symptoms improved. These findings are illustrated in Fig. 5A and B.

## Discussion

This retrospective study analyzed the clinical data of 259 SFTS patients aiming to identify the most effective combination of indicators for predicting the prognosis of SFTS. Multivariate logistic regression analysis revealed that advanced age, high viral load, AMS, and elevated PAU levels were independent risk factors for mortality in SFTS patients. As the first study to comprehensively assess PAU’s prognostic utility in SFTS, our findings demonstrate its robust predictive capability, offering a novel approach for early clinical risk assessment and outcome prediction in SFTS management.The PAU index is calculated as PCT × BUN/ALB. This novel composite marker integrates three biomarkers with well-established clinical significance: PCT, reflecting systemic inflammatory response and cytokine storm severity [14]; ALB, indicating liver synthetic function, nutritional status, and vascular endothelial integrity [15]; and BUN, representing renal function and protein metabolism status [16]. By combining these parameters, PAU enables a comprehensive assessment of both “inflammatory activation” and “multiorgan dysfunction”—two central pathophysiological features of SFTS—resulting in enhanced predictive performance (AUC: 0.810).

Notably, the elevation of PCT in SFTS patients warrants further investigation. Although viral infections typically do not induce marked increases in PCT, this study observed significantly higher levels in non-survivors compared to survivors. This may be attributed to the intense cytokine storm triggered by Severe Fever with Thrombocytopenia Syndrome Virus (SFTSV), which could directly stimulate PCT production; however, contributions from secondary bacterial infection or impaired hepatic and renal clearance cannot be excluded [17–18]. Reduced ALB levels reflect suppressed hepatic synthesis due to viral infection, increased vascular permeability leading to leakage, and accelerated catabolism under stress conditions [19–21]. Elevated BUN indicates renal impairment, potentially resulting from prerenal causes such as direct viral effects, inflammatory mediator-induced injury, or hypovolemia [22–25]. Thus, PAU captures multiple interrelated pathological processes—including inflammation, hepatic dysfunction, malnutrition, and renal failure—offering a more integrative approach to disease evaluation.

The predictive accuracy of PAU was significantly superior to that of conventional inflammatory indices such as NLR (AUC: 0.563) and PLR (AUC: 0.553). Moreover, it outperformed liver and kidney-related indicators including the AST/ALT ratio (AUC: 0.728) and the BAR (AUC: 0.767). The biological plausibility of this advantage lies in the inclusion of PCT—an essential marker of inflammation—alongside markers of organ damage. Given that fatal outcomes in SFTS result from the synergistic effects of direct viral injury, excessive immune response, and secondary multiorgan failure [26–27], PAU provides a more holistic assessment by simultaneously evaluating “inflammatory intensity” and “organ dysfunction,” likely explaining its superior prognostic value.

The optimal PAU cutoff value was determined to be 0.06 µmol/L, with a sensitivity of 75.7% and specificity of 73.0%. These findings have important implications for clinical practice. First, PAU calculation relies solely on routine laboratory tests available upon admission, making it feasible for implementation even in primary healthcare settings. A PAU ≥ 0.06 µmol/L at admission facilitates early identification of high-risk patients, prompting clinicians to consider intensified monitoring or earlier transfer to intensive care units. Second, elevated PAU suggests a state of “hyperinflammation concurrent with multiorgan deterioration,” supporting the consideration of targeted interventions. For instance, in patients with high PAU, beyond standard supportive therapy, enhanced organ support and evaluation for immunomodulatory treatment to mitigate cytokine storm should be prioritized. Furthermore, Kaplan-Meier survival analysis demonstrated that elevated PAU was significantly associated with poorer prognosis. Dynamic monitoring revealed decreasing PAU levels in survivors coinciding with clinical improvement, whereas non-survivors exhibited persistently rising values. This indicates that PAU may serve not only as an initial risk stratification tool but also as a potential marker for monitoring disease progression and therapeutic response.

Beyond single-marker analysis, we developed a logistic regression model incorporating age, viral load, AMS, and PAU, achieving an AUC of 0.897. Internal validation via bootstrapping (1,000 resamples, MAE = 0.012) showed minimal overfitting, with the calibration curve closely aligning with the ideal diagonal, indicating good model stability and clinical applicability.

This study has several limitations. First, its single-center, retrospective design introduces inherent selection bias and potential confounding, possibly affecting generalizability. Second, missing laboratory data for some patients were addressed through interpolation, which may impact result robustness. Third, dynamic changes in PAU over the disease course were not systematically assessed, limiting conclusions regarding its utility in longitudinal monitoring or treatment efficacy evaluation. Fourth, although patients with co-infections or unclear primary diagnoses were excluded, post-admission bacterial infections and subsequent antibiotic use might elevate PCT levels, potentially influencing PAU interpretation. Last, the absence of external validation limits the broader application of the prediction model. Future studies should include multicenter prospective designs, establish external cohorts, longitudinally collect PAU and other biomarker data, validate their predictive and monitoring capabilities, and explore their role in guiding individualized therapeutic strategies.

## Conclusion

In summary, PAU, as a novel composite biomarker, integrates indicators of inflammation and organ dysfunction, offering a reliable and superior alternative to traditional prognostic tools in SFTS. Its strong predictive performance and alignment with core disease mechanisms suggest that PAU holds promise as a practical tool for clinical risk stratification and management. Even when incorporated into a prediction model alongside established risk factors—advanced age, AMS, and high viral load—PAU remained the strongest independent contributor, highlighting its potential as a cornerstone of future prognostic scoring systems. This finding not only advances methodological approaches to SFTS prognosis but also offers valuable insights for outcome prediction in other severe infectious diseases.

## Supplementary Information

Below is the link to the electronic supplementary material.

Supplementary Material 1

## Data Availability

The datasets supporting the findings of this study are obtainable from the corresponding author upon reasonable request.

## Abbreviations

SFTS: Severe fever with thrombocytopenia syndrome
NBV: Novel bunyavirus
PAU: Procalcitonin/albumin to urea nitrogen ratio
NLR: Neutrophil-to-lymphocyte ratio
PLR: Platelet-to-lymphocyte ratio
PAR: Platelet-to-albumin ratio
BAR: Blood urea nitrogen-to-albumin ratio
DM: Diabetes mellitus
HTN: Hypertension
CHD: Coronary heart disease
CVD: Cerebrovascular disease
WBC: White blood cell count
NEUT: Neutrophil count
LYMPH: Lymphocyte count
PLT: Platelet count
ALB: Serum albumin
ALT: Alanine aminotransferase
AST: Aspartate aminotransferase
LDH: Lactate dehydrogenase
BUN: Blood urea nitrogen
PCT: Procalcitonin
APTT: Activated partial thromboplastin time
PT: Prothrombin time
AMS: Altered mental status
ROC: Receiver operating characteristic
SD: Standard deviation
IQR: Interquartile range
SFTSV: Severe fever with thrombocytopenia syndrome virus
IFN-γ: Interferon-γ
AUC: Area under the curve
CI: Confidence interval
OR: Odds ratio
MAE: Mean absolute error
ICU: Intensive care unit
CRRT: Continuous renal replacement therapy
