# Supplementary material for: Procalcitonin/albumin to urea nitrogen ratio: a novel prognostic indicator for severe fever with thrombocytopenia syndrome
Source: BMC Infect Dis. 2026 Jan 29;26:431. doi: 10.1186/s12879-026-12736-6 (PMC12924486; doi:10.1186/s12879-026-12736-6)
Supplement: Supplementary file 1 — Supplementary Material 1 [file 12879_2026_12736_MOESM1_ESM.docx]

Table S1 Organ Support Status

| Characteristics | Survival(n=172) | Death (n=87) | t/z/χ² value | PValue |
| --- | --- | --- | --- | --- |
| ICU transfer, n (%) | 26(15.11) | 49(56.32) | 47.685 | <0.001 |
| Invasive mechanical ventilation *, n (%) | 9(5.23)* | 26(29.88)* | 30.044 | <0.001 |
| Continuous pumping of catecholamines *, n (%) | 14(8.14)* | 33(37.93)* | 34.522 | <0.001 |
| CRRT*, n (%) | 1(0.58)* | 9(10.34)* | 14.837 | <0.001 |

Abbreviations:ICU, intensive care unit; CRRT, continuous renal replacement therapy.

*All invasive mechanical ventilation, continuous pumping of catecholamines and CRRT occurred in ICU patients. In general wards, only basic monitoring, oxygen therapy or non-invasive antihypertensive drugs are provided, and the above-mentioned advanced life support is not carried out. Therapeutic support measures were only compared between groups and were not included in the regression model.

Table S2: Psychological Intervention and Antibacterial Management

| Characteristics | Survival(n=172) | Death (n=87) | t/z/χ² value | P Value |
| --- | --- | --- | --- | --- |
| Psychological consultation, n (%) | 3(1.74) | 6(6.89) | 4.573 | 0.032 |
| Secondary infection, n (%) | 38(22.09) | 31(35.63) | 5.419 | 0.020 |
| Empirical antibiotic use, n (%) | 58(33.72) | 68(78.16) | 45.674 | <0.001 |

Psychological consultation: For those with delirium or abnormal emotions; Antibiotics: Piperacillin-tazobactam/meropenem should be administered immediately after diagnosis or when secondary infection occurs. Psychological intervention and antibacterial management indicators are intermediate variables, only used for description and not included in univariate/multivariate analysis.
